# Candidemia Risk Prediction (CanDETEC) Model for Patients With Malignancy: Model Development and Validation in a Single-Center Retrospective Study

**DOI:** 10.2196/24651

**Published:** 2021-07-26

**Authors:** Junsang Yoo, Si-Ho Kim, Sujeong Hur, Juhyung Ha, Kyungmin Huh, Won Chul Cha

**Affiliations:** 1 Department of Nursing College of Nursing Sahmyook University Seoul Republic of Korea; 2 Division of Infectious Disease Samsung Changwon Hospital Sungkyunkwan University School of Medicine Changwon Republic of Korea; 3 Department of Patient Experience Management Samsung Medical Center Seoul Republic of Korea; 4 Department of Digital Health Samsung Advanced Institute for Health Sciences & Technology Sungkyunkwan University Seoul Republic of Korea; 5 Department of Computer Science Indiana University Bloomington Bloomington, IN United States; 6 Division of Infectious Disease Samsung Medical Center Sungkyunkwan University School of Medicine Seoul Republic of Korea; 7 Department of Emergency Medicine Samsung Medical Center Sungkyunkwan University School of Medicine Seoul Republic of Korea; 8 Digital Innovation Center Samsung Medical Center Seoul Republic of Korea

**Keywords:** candidemia, precision medicine, supervised machine learning, decision support systems, clinical, infection control, decision support, machine learning, development, validation, prediction, risk, model

## Abstract

**Background:**

Appropriate empirical treatment for candidemia is associated with reduced mortality; however, the timely diagnosis of candidemia in patients with sepsis remains poor.

**Objective:**

We aimed to use machine learning algorithms to develop and validate a candidemia prediction model for patients with cancer.

**Methods:**

We conducted a single-center retrospective study using the cancer registry of a tertiary academic hospital. Adult patients diagnosed with malignancies between January 2010 and December 2018 were included. Our study outcome was the prediction of candidemia events. A stratified undersampling method was used to extract control data for algorithm learning. Multiple models were developed—a combination of 4 variable groups and 5 algorithms (auto-machine learning, deep neural network, gradient boosting, logistic regression, and random forest). The model with the largest area under the receiver operating characteristic curve (AUROC) was selected as the *Candida* detection (CanDETEC) model after comparing its performance indexes with those of the Candida Score Model.

**Results:**

From a total of 273,380 blood cultures from 186,404 registered patients with cancer, we extracted 501 records of candidemia events and 2000 records as control data. Performance among the different models varied (AUROC 0.771- 0.889), with all models demonstrating superior performance to that of the Candida Score (AUROC 0.677). The random forest model performed the best (AUROC 0.889, 95% CI 0.888-0.889); therefore, it was selected as the CanDETEC model.

**Conclusions:**

The CanDETEC model predicted candidemia in patients with cancer with high discriminative power. This algorithm could be used for the timely diagnosis and appropriate empirical treatment of candidemia.

## Introduction

Candidemia is a representative nosocomial bloodstream infection that contributes to the mortality of immunocompromised patients; it has been shown to occur in 3% of patients in intensive care and 20% of immunosuppressed patients [1]. In addition, owing to a compromised immunity from chemotherapy or malignancy itself, patients with cancer have been reported as the most vulnerable hosts to candidemia [2-4].

Significant mortality has been reported over several decades. In studies from the 1980s, the mortality rate in patients with cancer found to have candidemia exceeded 50% [5-7]. High mortality rates, ranging from 30% to 51%, have also been reported in studies after 2010 [3,4,8]. The mortality rate of candidemia was significantly higher than that of bacteremia [3].

Early empirical treatment is important for patients with candidemia. A retrospective study [8] showed that patients with candidemia whose antifungal treatment was initiated 12 hours after onset of candidemia had a hospital mortality rate twice that of patients whose antifungal treatment was initiated within 12 hours of onset. Despite evidence on the need for early treatment of candidemia, only a small number of patients, receive timely antifungal treatment because of the difficulty of early diagnosis [9].

There has been an unmet clinical need—the coexistence of timeliness, high reliability, and cost-effectiveness in candidemia diagnosis. Blood culture is the reference standard for the candidemia diagnosis [10]. Due to its inherent nature, obtaining results can take a median of 2 to 3 days. Thus, this delay constitutes one challenge in the problem of timely diagnosis [11]. Multiple statistical models, such as the Candida Score, have been developed for the early prediction of candidemia [12-14]. However, such models have neither been tested on unseen data sets during development nor shown consistent performance in subsequent external validation studies [15-17]. The new T2Candida molecular test, which combines magnetic resonance with molecular diagnostics, is useful for the detection of candidemia in a very short amount of time and with high accuracy [18]; however, the high cost of the T2Candida molecular test is a barrier to its wide application in clinical practice [19,20].

Electronic health records allow efficient extraction and integration of clinical data, and the development of machine learning algorithms for critical care has been vigorously researched [21,22]. We aimed to develop a candidemia prediction model for patients with cancer using machine learning algorithms.

## Methods

### Study Population

**Figure 1:**
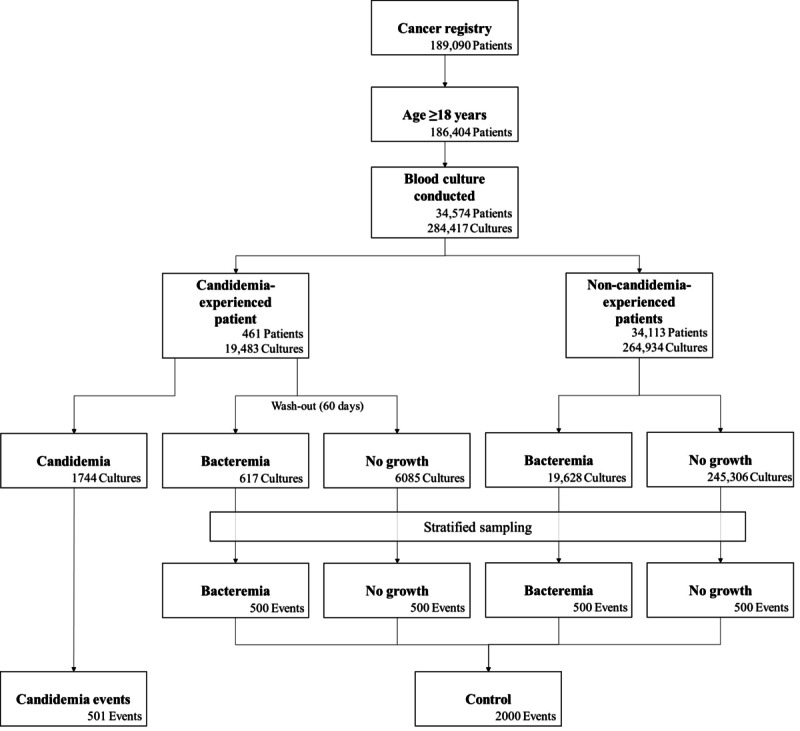
Eligibility process diagram.

This study was conducted in a 1950-bed tertiary academic single hospital in Seoul, Republic of Korea. The study population included adult patients (≥18 years old) diagnosed with a malignancy and from whom blood cultures had been obtained between January 2010 and December 2018 after diagnosis. The data set used in this analysis was extracted from the cancer registry and clinical data warehouse of the study site. The selection process is shown in Figure 1.

### Outcome

We defined *candidemia event* as a positive culture for any *Candida* species in more than one blood sample. If candidemia was detected in a follow-up culture within 7 days, the subsequent event was merged with previous candidemia events. Because the algorithm was designed to predict candidemia events at the time of blood culture extraction, data were processed at the level of each event rather than at the patient level.

A data set with a low candidemia prevalence can cause difficulties in model training [23,24]. To solve this problem, stratified undersampling was conducted at a 1:4 ratio in 4 different subsets (used as a control data): (1) events of bacteremia in patients who experienced candidemia; (2) events of negative blood culture in patients who experienced candidemia; (3) events of bacteremia in patients who had not experienced candidemia; and (4) events of negative blood culture in patients who had not experienced candidemia. In the control subsets with patients who had experienced candidemia, a 60-day wash-out period was imposed from the day of onset of the candidemia event.

### Model Development

#### Stage 1: Feature Selection and Preprocessing

**Figure 2:**
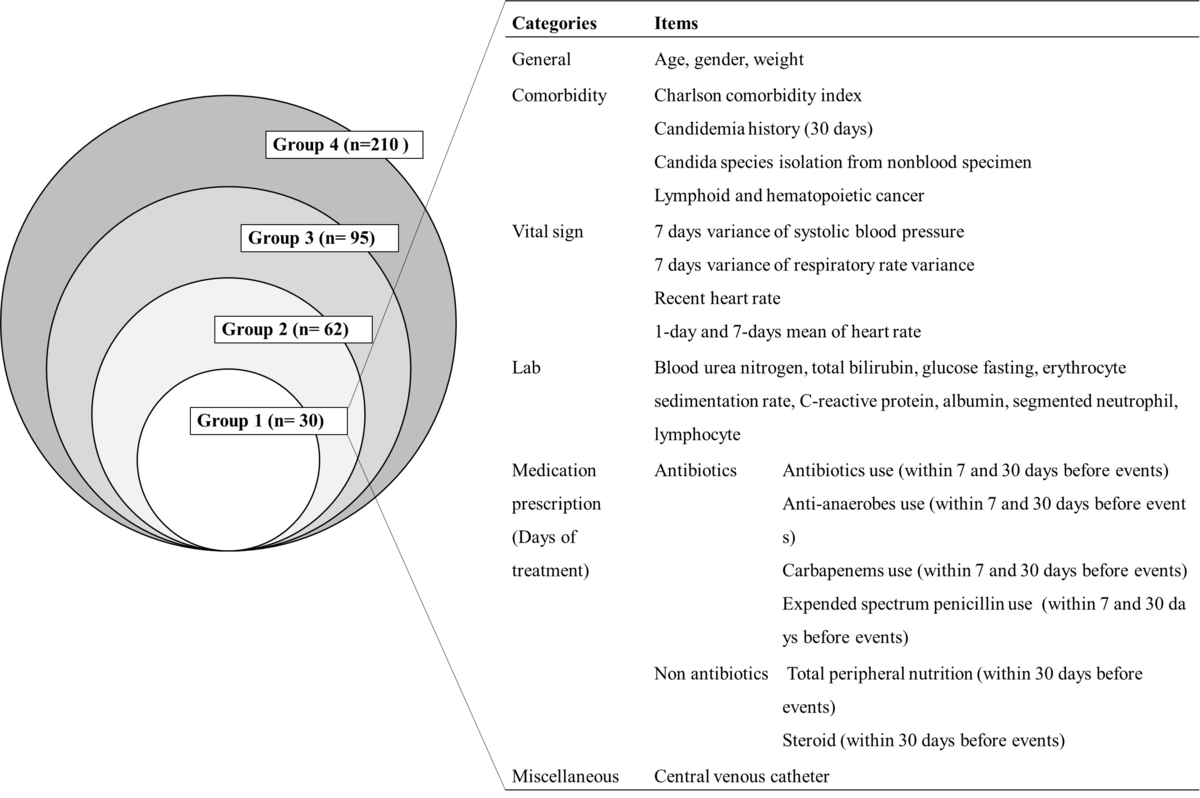
Variable groups used for training algorithms and a detailed list of variable group 1.

Upon review of clinical-domain literature on candidemia risk factors, we identified 210 variables [2,13,14,25-27] that have been widely used in the development of machine learning algorithms in other clinical fields. We extracted data for these variables from the electronic health records of the study site. Each variable was classified into 4 groups based on variable importance, clinical importance, auto-extractability, and missing rate (Figure 2 and Multimedia Appendix 1). We gradually eliminated features: variable group 4 had 210 variables, whereas variable group 1 (higher variable importance, higher clinical importance, better extractability, and less missing values) had only 30 variables. In order to prevent the algorithm from learning from postdiagnostic data, we removed candidemia diagnostic code and antifungal agent prescription information from the input data.

To impute missing data, we used 2 serial methods. We used the carry-forward method to fill empty bins with the most recent value. This method reflects the workflow of the clinician in recording new data as the patients’ condition changes; it is also easy and simple. In the case of missing values that were not imputed by the carry-forward method, average values were used. All numerical variables were normalized.

#### Stage 2: Data Partitioning

The data set was divided into training and test sets (7:3 ratio) using stratified sampling, matched by case–control group, binned age, Charlson comorbidity index, and sex.

#### Stage 3: Model Development

A total of 20 models were developed using a combination of 5 algorithms (logistic regression, deep neural network, random forest, gradient boosting, and automated machine learning) algorithms and 4 variable groups. For each model, 100 different development trials were conducted by changing random seeds to prevent selective performance reporting in the subsequent model evaluation stage. We used 2 methods for model selection and parameter optimization—an automated machine learning tool called the tree-based pipeline optimization tool [28], which helps identify the best prediction model and the best parameters for each variable group by using genetic algorithms and cross-validated performance on the training set (Multimedia Appendix 2), and a simple grid search, which is used to pick the parameters with the best performance.

#### Stage 4. Model Evaluation

Each model was evaluated using a test set (unseen data). Area under the receiver operating characteristic curve (AUROC), sensitivity, specificity, positive predictive value, negative predictive value, and F1 score were used to measure performance. Under the assumption that the algorithm was to be used as a screening tool, sensitivity >0.90 with the highest F1 score, was determined as the threshold for classifying the risk group. One-way analysis of variance was conducted to compare AUROC values. Statistical significance was set at .05. Subsequently, the Bonferroni test was conducted with α=.0025 for each of the 20 outcomes combining the algorithm types and variable groups.

The model with the highest AUROC was selected as the Candida detection (CanDETEC) algorithm. To examine how well the predicted risk correlated with the observed risk, we generated a linear regression model using 10 bins based on the predicted risk of the CanDETEC model. In general, if the coefficients and intercept of the linear regression model were close to 1 and 0, respectively, the model was considered well calibrated.

Candida Score [13], a traditional statistical candidemia prediction model, was used as the standard. We compared the CanDETEC model and the rounded Candida Score: 1 × (total parenteral nutrition) + 1 × (surgery) + 1 × (multifocal candida colonization) + 2 × (severe sepsis) [13]. Performance indexes were calculated for Candida Score >3. We also employed a net benefit index to compare both models as well as to determine whether the chosen threshold could have beneficial clinical implications.

### Statistical Programming

We used R (version 3.6.2; The R Project) and Python (version 3.6.8) for data preprocessing, statistical analysis, visualization, development, and validation of machine learning algorithms [29,30]. The sample preprocessed data set (20 records) and code for developing our models are available [31].

## Results

### Study Population

A total of 186,404 adult patients were in the cancer registry. A total of 273,380 blood cultures were obtained from 34,574 patients after cancer diagnosis, with 1744 (0.6%) *Candida* species isolated from blood cultures. A total of 501 candidemia events were identified in 461 patients. The most predominant species of Candida were *C albicans* (164/501, 32.7%), *C tropicalis* (162/501, 32.3%), and *C glabrata* (96/501, 19.2%) (Multimedia Appendix 3). We found 40 repeat candidemia events, and the median interval between events was 21.4 days. (IQR 14.5-57.4 days). Of the 271,636 blood cultures with a negative candidemia result, 2000 were extracted as control data.

Hematologic malignancy was the most common malignancy (Table 1). Several clinical factors were significantly associated with case events. Patients with candidemia received longer treatment with steroids, parenteral nutrition, and antibiotics within 30 days before candidemia. Furthermore, antianaerobic therapy (mean 10.1 days) was twice as long (*P*<.001) in those with candidemia than that in controls (5.5 days). In addition, patients with candidemia had higher C-reactive protein, bilirubin, fasting glucose, blood urea nitrogen, and lactic acid levels. The 30-day all-cause mortality rate was significantly higher (*P*<.001) among those with candidemia (264/501, 52.7%) than among controls (303/2000, 15.2%). There was no difference (*P*=.18) in the use of antifungal agents in candidemia (70/501, 14.0%) and control (233/2000, 11.6%) records (Table 2).

**Table 1:** General characteristics of the study data set.

Characteristic				Candidemia events (n=501)		Control (n=2000)		*P* value
**Sex, n (%)**								.32
	Male			297 (59.3)		1237 (61.9)		
	Female							
Age (years), mean (SD)				59.5 (14.4)		55.9 (14.9)		<.001
Hospital stay before culture (days), mean (SD)				23.0 (25.0)		22.2 (65.4)		.66
**Comorbidity, n (%)**								
	Hepatic disease			60 (12.0)		298 (14.9)		.11
	Cardiovascular disease			68 (13.6)		203 (10.2)		.03
	Endocrine disease			54 (10.8)		198 (9.9)		.62
	Digestive disease			36 (7.2)		117 (5.8)		.31
	Respiratory disease			20 (4.0)		79 (4.0)		>.999
	Other disease			11 (2.2)		38 (1.9)		.80
Charlson Comorbidity Index, mean (SD)				4.7 (2.3)		4.2 (2.2)		<.001
**Cancer origin site, n (%)**								
	Lymphoid or hematopoietic			231 (46.1)		1116 (55.8)		<.001
	Digestive			153 (30.5)		551 (27.6)		.20
	Respiratory			43 (8.6)		113 (5.7)		.02
	Female genital			23 (4.6)		49 (2.5)		.02
	Other			38 (7.6)		122 (6.1)		.27
	Multiple primary			44 (8.8)		235 (11.8)		.07
Metastatic lymph nodes, mean (SD)				5.1 (12.2)		3.5 (9.9)		.007
**Medication (days of therapy during 30 days before candidemia), mean (SD)**								
	Steroid			8.6 (10.2)		5.8 (9.0)		<.001
	Immunosuppressant			1.4 (5.7)		1.6 (5.1)		.43
	Total peripheral nutrition			4.6 (7.8)		2.5 (5.8)		<.001
	**Antibiotic use**			16.9 (9.3)		15.3 (9.8)		.002
		Antianaerobic	10.1 (8.6)		5.5 (8.2)		<.001	
		Broad spectrum cephalosporine: 3rd generation	3.5 (4.8)		2.6 (4.5)		<.001	
		Carbapenem	4.8 (5.8)		2.5 (5.4)		<.001	
		Extended spectrum penicillin	4.8 (5.8)		2.4 (5.1)		<.001	
		Glycopeptide	3.8 (5.0)		1.8 (4.1)		<.001	
**Vitals, mean (SD)**								
	Systolic blood pressure (mmHg)			118.9 (23.4)		117.5 (20.8)		.21
	Diastolic blood pressure (mmHg)			68.5 (14.0)		68.6 (13.4)		.89
	Heart rate (bpm)			110.1 (21.3)		103.3 (20.6)		<.001
	Respiratory rate (brpm)			21.1 (5.1)		20.1 (4.2)		<.001
	Body temperature (°C)			37.2 (1.0)		37.6 (1.0)		<.001
	Peripheral capillary oxygen saturation (%)			96.2 (6.1)		97.0 (4.2)		.03
**Laboratory work-up, mean (SD)**								
	**Complete blood count**							
		White blood cell count, blood (10^3^/µL)	8.3 (11.2)		6.8 (18.0)		.02	
		Hemoglobin, blood (g/dL)	9.4 (1.4)		9.7 (1.8)		<.001	
		Platelet count, blood (10³/µl)	90.1 (103.1)		104.7 (151.0)		.01	
		Segmented neutrophil (%)	62.3 (35.3)		54.0 (36.2)		<.001	
		Absolute neutrophil count (10³/µL)	7.2 (10.3)		4.9 (7.5)		<.001	
		Absolute lymphocyte count (10³/µL)	0.5 (0.7)		0.7 (1.6)		.009	
	**Acute phase reactants**							
		Erythrocyte sedimentation rate (mm/h)	41.6 (36.8)		48.4 (34.3)		.001	
		C-reactive protein (mg/dL)	11.2 (9.0)		8.6 (7.9)		<.001	
		Procalcitonin, quantitative (ng/mL)	6.3 (16.3)		3.9 (14.4)		.07	
	**Coagulation**							
		Prothrombin time (international normalized ratio)	1.4 (0.5)		1.3 (0.5)		<.001	
	**Chemistry**							
		Total protein (g/dL)	5.3 (1.0)		5.8 (1.1)		<.001	
		Albumin (g/dL)	2.9 (0.5)		3.4 (0.6)		<.001	
		Globulin (g/dL)	2.3 (0.8)		2.5 (0.8)		.001	
		Cholesterol (mg/dL)	129.1 (64.8)		138.4 (51.3)		.006	
		Total bilirubin (mg/dL)	5.5 (9.1)		2.4 (4.7)		<.001	
		Alkaline phosphatase (U/L)	183.1 (178.2)		157.9 (217.1)		.007	
		Glucose fasting (mg/dL)	158.7 (71.2)		139.2 (59.2)		<.001	
		Blood urea nitrogen (mg/dL)	34.1 (25.3)		22.8 (17.4)		<.001	
		Creatinine (mg/dL)	1.1 (1.0)		1.0 (1.0)		.01	
		Uric acid (mg/dL)	3.5 (2.3)		3.6 (2.4)		.59	
		Calcium (mg/dL)	8.3 (0.8)		8.5 (0.8)		<.001	
		Phosphorus (mg/dL)	3.1 (1.2)		3.2 (1.1)		.03	
		Lactic acid (mmol/L)	2.9 (2.8)		2.2 (2.3)		<.001	
**Other risk factors, n (%)**								
	Candidemia history (within 30 days)			26 (5.2)		0 (0.0)		<.001
	Candida isolation from nonblood specimen (within 30 days)			110 (22.0)		132 (6.6)		<.001
	Central line inserted			223 (44.5)		854 (42.7)		.50
	Major operation (within 30 days)			78 (15.6)		219 (10.9)		.005

**Table 2:** Outcome characteristics of the study population.

Characteristic			Candida event (n=501), n (%)		Control (n=2000), n (%)		*P* value
**Prescription of antifungal agents**							
	On the day of the blood culture	70 (14.0)		233 (11.6)		.18	
	Within 3 days after blood culture	277 (55.3)		770 (38.5)		<.001	
30-Day all-cause mortality			264 (52.7)		303 (15.2)		<.001

### Model Evaluation

**Figure 3:**
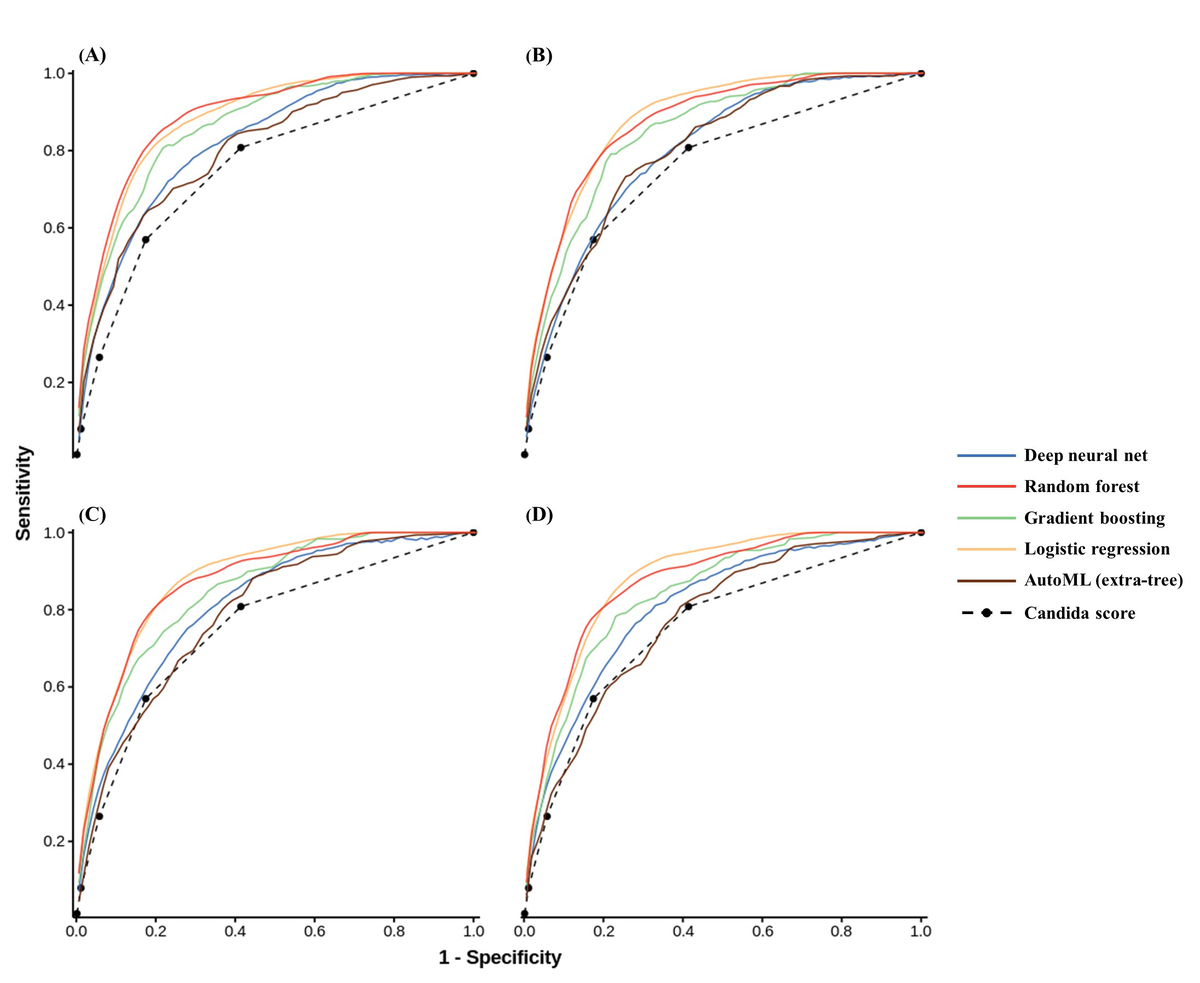
Area under the receiver operating characteristic curve of the developed models by algorithm type and variables groups: (A) Group 1, (B) Group 2, (C) Group 3, and (D) Group 4. AutoML: automated machine learning.

The best model was the random forest model trained using variable group 1, which was selected as the CanDETEC algorithm (Table 3; Multimedia Appendix 4); the lowest performing model was the logistic regression model trained using variable group 4. Receiver operating characteristic curves are shown in Figure 3. The tree-based pipeline optimization tool algorithm returned an extra-tree model, which showed the highest performance at the specified cut-off. Among the 30 auto-extractable variables, 5 variables showed the highest significance (*P*<.001) in the prediction of candidemia: blood urea nitrogen level, 7-day variance of respiratory rate, total bilirubin level, 7-day variance of systolic blood pressure, and body weight (Multimedia Appendix 5).

**Table 3:** Model performances by algorithm and variable group.

Algorithm and variable group			AUROC (95% CI)		Sensitivity (95% CI)		Specificity (95% CI)		Positive predictive value (95% CI)		Negative predictive value (95% CI)		F1 score (95% CI)
**Logistic regression**													
	1	0.802 (0.802-0.802)		0.907 (0.907-0.907)		0.451 (0.451-0.451)		0.293 (0.293-0.293)		0.951 (0.951-0.951)		0.443 (0.443-0.443)	
	2	0.8 (0.8-0.8)		0.901 (0.901-0.901)		0.479 (0.479-0.479)		0.303 (0.303-0.303)		0.95 (0.95-0.95)		0.453 (0.453-0.453)	
	3	0.788 (0.788-0.788)		0.901 (0.901-0.901)		0.521 (0.521-0.521)		0.321 (0.321-0.321)		0.954 (0.954-0.954)		0.473 (0.473-0.473)	
	4	0.771 (0.771-0.771)		0.901 (0.901-0.901)		0.473 (0.473-0.473)		0.3 (0.3-0.3)		0.95 (0.95-0.95)		0.45 (0.45-0.45)	
**Random forest**													
	1^a^	0.889 (0.888-0.889)		0.901 (0.901-0.902)		0.722 (0.719-0.724)		0.449 (0.447-0.451)		0.967 (0.967-0.967)		0.599 (0.597-0.601)	
	2	0.872 (0.872-0.873)		0.901 (0.901-0.902)		0.669 (0.667-0.672)		0.407 (0.405-0.409)		0.964 (0.964-0.964)		0.56 (0.559-0.562)	
	3	0.869 (0.869-0.87)		0.902 (0.901-0.902)		0.642 (0.639-0.645)		0.388 (0.386-0.39)		0.963 (0.963-0.963)		0.542 (0.54-0.544)	
	4	0.87 (0.87-0.871)		0.901 (0.901-0.901)		0.669 (0.666-0.672)		0.407 (0.404-0.409)		0.964 (0.964-0.964)		0.56 (0.558-0.562)	
**Extra tree^b^**													
	1	0.881 (0.88-0.881)		0.901 (0.901-0.902)		0.67 (0.665-0.675)		0.408 (0.404-0.412)		0.964 (0.964-0.965)		0.561 (0.558-0.565)	
	2	0.882 (0.881-0.882)		0.902 (0.901-0.902)		0.715 (0.711-0.719)		0.443 (0.44-0.447)		0.967 (0.966-0.967)		0.594 (0.591-0.597)	
	3	0.879 (0.879-0.88)		0.901 (0.901-0.901)		0.708 (0.703-0.713)		0.438 (0.434-0.442)		0.966 (0.966-0.966)		0.589 (0.585-0.592)	
	4	0.879 (0.878-0.879)		0.902 (0.901-0.903)		0.717 (0.713-0.721)		0.445 (0.442-0.448)		0.967 (0.967-0.967)		0.596 (0.593-0.599)	
**Gradient boosting**													
	1	0.861 (0.861-0.862)		0.901 (0.901-0.901)		0.621 (0.621-0.621)		0.374 (0.374-0.374)		0.961 (0.961-0.961)		0.528 (0.528-0.528)	
	2	0.847 (0.847-0.847)		0.901 (0.901-0.901)		0.593 (0.592-0.593)		0.357 (0.357-0.357)		0.96 (0.96-0.96)		0.511 (0.511-0.512)	
	3	0.846 (0.846-0.846)		0.901 (0.901-0.901)		0.573 (0.573-0.573)		0.346 (0.346-0.346)		0.958 (0.958-0.958)		0.5 (0.5-0.5)	
	4	0.839 (0.839-0.839)		0.901 (0.901-0.901)		0.562 (0.562-0.563)		0.341 (0.341-0.341)		0.958 (0.957-0.958)		0.495 (0.494-0.495)	
**Deep neural network**													
	1	0.82 (0.818-0.821)		0.901 (0.901-0.902)		0.499 (0.494-0.504)		0.312 (0.309-0.314)		0.953 (0.952-0.953)		0.463 (0.461-0.466)	
	2	0.799 (0.797-0.801)		0.902 (0.901-0.902)		0.505 (0.5-0.51)		0.314 (0.312-0.317)		0.953 (0.953-0.954)		0.466 (0.464-0.469)	
	3	0.809 (0.807-0.811)		0.902 (0.901-0.902)		0.525 (0.516-0.534)		0.324 (0.32-0.327)		0.954 (0.953-0.956)		0.476 (0.472-0.48)	
	4	0.807 (0.804-0.81)		0.901 (0.901-0.902)		0.508 (0.499-0.517)		0.316 (0.313-0.32)		0.953 (0.952-0.954)		0.468 (0.464-0.472)	

^a^CanDETEC model.

^b^Automated machine learning.

### CanDETEC Model

**Figure 4:**
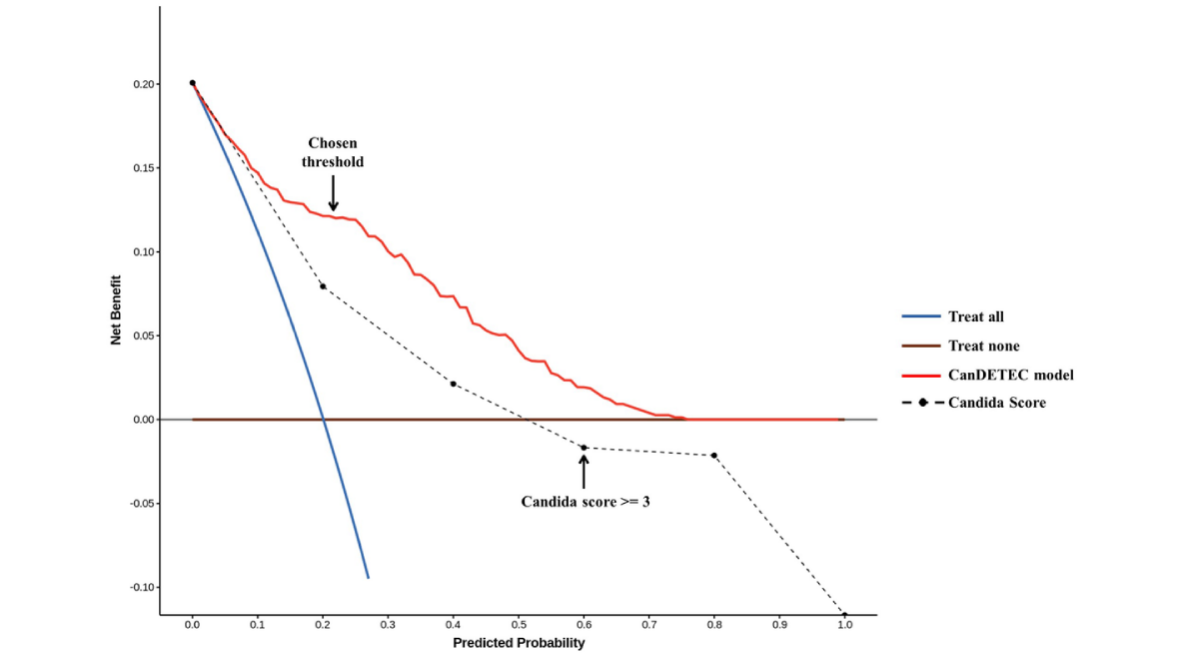
Decision curve of the CanDETEC model. Arrow indicates the threshold to determine candidemia high risk group.

**Figure 5:**
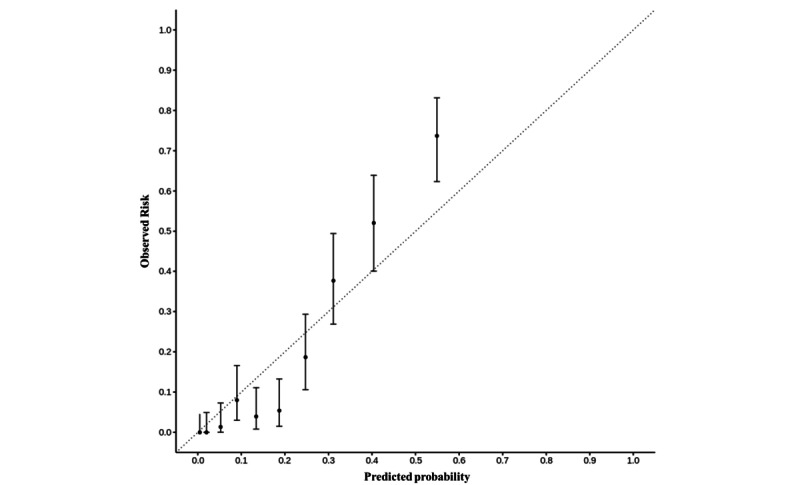
Calibration plot of the CanDETEC model. A point represents mean decile grouped by predicted probability. Error bars represent 95% confidence interval.

The threshold for categorizing the candidemia risk group was set at 0.216 by a predefined condition (sensitivity >0.90 and highest F1 score). At this cut-off point, the calculated net benefit was 0.121 (Figure 4). This threshold not only had a greater net benefit than that of treating all or none of the patients but was also close to the point showing the most significant net benefit. The coefficients, intercept, and *R*^2^ of the linear regression model for evaluating calibration were 1.393, 0.078, and 0.93, respectively. These indexes show that the CanDETEC model was acceptably calibrated (Figure 5).

The diagnostic performance of the Candida Score, when the cut-off point was defined as ≥3, was worse than that of all other models (AUROC 0.677, F1 score 0.354, sensitivity 0.265, specificity 0.942, positive predictive value 0.533, negative predictive value 0.836).

## Discussion

### Principal Results

We developed a novel algorithm, called CanDETEC, to predict candidemia among patients with cancer with suspected sepsis. Given that this model only requires auto-extractable variables with low missing rates, it can be easily applied in clinical settings to help clinicians with decision making in prescribing empirical antifungal agents.

Our CanDETEC model was developed using variables that reflect the dynamic status of patients with cancer. It seems that adopting these variables to the CanDETEC model will contribute to providing timely decision support when this algorithm applied to a real clinical setting. For example, the variable importance of variance of the respiration rate was second highest (Multimedia Appendix 5). Therefore, this model could also be used in real time.

### Comparison With Prior Work

The CanDETEC algorithm can be used to support clinicians’ decision-making process in providing appropriate empirical treatment for candidemia in patients with cancer. Although candidemia contributes to the mortality of patients diagnosed with malignancies, the timely diagnosis of candidemia remains clinically challenging [8]. Given its low prevalence, busy clinicians often overlook candidemia as a possible cause of infection. Only 14.0% of patients (70/501) in our data set with candidemia had received empirical antifungal agents, and the 30-day all-cause mortality rate exceeded 50% (264/501, 52.7%), which is consistent with outcomes in previous studies [3,4,8,9]. Furthermore, infectious disease diagnostic processes that are currently used have a high cognitive burden as they require the collection and calculation of several complicated patient information variables [32]. Because the CanDETEC algorithm can be used without the clinician’s subjective judgment, our model has the potential to support physicians’ decision-making process with minimal additional workload.

Current tools for predicting candidemia have major limitations. Although the Candida Score is the most widely used tool to predict invasive candidemia, its sensitivity was reported to be only 37% in a recent external validation study [16], which was lower than the 81% in the original study [13]. This might have been a result of differences in the patient population. Static variables may also hinder the appropriate prediction of candidemia because they cannot appropriately reflect changes in the clinical status of patients over time. Not surprisingly, the Candida Score showed relatively low diagnostic performance in our study (AUROC 0.677).

### Limitations

Our study has several limitations. First, CanDETEC was designed to predict candidemia when blood cultures were performed; however, the Candida Score was originally designed to predict invasive candidiasis, including candidemia. Thus, a comparison of diagnostic performance between our model and should be interpreted with caution. Second, this was a single-center retrospective study. Further multicenter prospective studies are required for external validation and to prove the clinical efficacy of the CanDETEC model. Third, although we developed a model with clinically acceptable performance, we only applied basic machine learning, such as random forest and gradient boosting. Recently, more complex ensemble models have been developed, and they have presented better performance compared to that of basic machine learning models in other medical domains [33,34]. Therefore, a follow-up study employing a state-of-the-art model should be conducted to examine whether the performance of the CanDETEC model could be improved.

### Conclusions

Our CanDETEC model, to predict candidemia in patients with cancer, is expected to reduce the mortality of patients with malignancy by helping the clinician with decision making for timely antifungal treatment.

## Appendix

Multimedia Appendix 1 Variable group classification process.

Multimedia Appendix 2 Detailed model parameters.

Multimedia Appendix 3 Characteristics of candidemia events.

Multimedia Appendix 4 Bonferroni-corrected posthoc comparisons.

Multimedia Appendix 5 Variable importance of the CanDETEC model.

## Abbreviations

AUROC: area under the receiver operating characteristic curve
