# Correction: Candidemia Risk Prediction (CanDETEC) Model for Patients With Malignancy: Model Development and Validation in a Single-Center Retrospective Study

**DOI:** 10.2196/36385

**Published:** 2022-01-19

**Authors:** Junsang Yoo, Si-Ho Kim, Sujeong Hur, Juhyung Ha, Kyungmin Huh, Won Chul Cha

**Affiliations:** 1 Department of Nursing College of Nursing Sahmyook University Seoul Republic of Korea; 2 Division of Infectious Disease Samsung Changwon Hospital Sungkyunkwan University School of Medicine Changwon Republic of Korea; 3 Department of Patient Experience Management Samsung Medical Center Seoul Republic of Korea; 4 Department of Digital Health Samsung Advanced Institute for Health Sciences & Technology Sungkyunkwan University Seoul Republic of Korea; 5 Department of Computer Science Indiana University Bloomington Bloomington, IN United States; 6 Division of Infectious Disease Samsung Medical Center Sungkyunkwan University School of Medicine Seoul Republic of Korea; 7 Department of Emergency Medicine Samsung Medical Center Sungkyunkwan University School of Medicine Seoul Republic of Korea; 8 Digital Innovation Center Samsung Medical Center Seoul Republic of Korea

In “Candidemia Risk Prediction (CanDETEC) Model for Patients With Malignancy: Model Development and Validation in a Single-Center Retrospective Study” (JMIR Med Inform 2021;9(7):e24651), one error was noted.

Due to a system error, the ORCID number of author Sujeong Hur was incorrectly published as:

0000-0001-7763-8940

This has been corrected to:

0000-0003-1335-576X

The correction will appear in the online version of the paper on the JMIR Publications website on January 19, 2022, together with the publication of this correction notice. Because this was made after submission to PubMed, PubMed Central, and other full-text repositories, the corrected article has also been resubmitted to those repositories.

