# Supplementary material for: Candidemia Risk Prediction (CanDETEC) Model for Patients With Malignancy: Model Development and Validation in a Single-Center Retrospective Study
Source: JMIR Med Inform. 2021 Jul 26;9(7):e24651. doi: 10.2196/24651 (PMC8367162; doi:10.2196/24651)
Supplement: Multimedia Appendix 2 [file medinform_v9i7e24651_app2.docx]

Multimedia Appendix 2. Detailed developed model parameter.

| algorithm | Parameter | value |
| --- | --- | --- |
| Logistic regression | Variable group | 1 |
|  | Penalty (regularization) | L2 |
| Random forest | Variable group | 1 |
|  | N of trees | 100 |
|  | Split criterion | Gini index |
|  | Sampling method | Bootstrap conducted |
|  | Max number of feature | 5 |
| Auto-ML (TPOT) | Variable group | 2 |
|  | Generation | 3 |
|  | Population size | 100 |
|  | Cross validation | 5 |
|  | Scoring metrics | AUROC |
| Gradient Boosting | Variable group | 1 |
|  | Learning rate | 0.1 |
|  | Number of boosting stages | 100 |
| DNN | Variable group | 1 |
|  | Activation  (hidden layer) | ReLU |
|  | Activation  (output layer) | Sigmoid |
|  | Hidden layer sizes | 1000, 500, 100 |
|  | Local random seed | Not specified |
|  | Epochs | 100 with early stopping |
|  | Epsilon | Not specified |
|  | Rho | Not specified |
|  | L1 | Not specified |
|  | L2 | Not specified |
|  | optimizer | Adam with learning rate 10-4 |
|  | loss | Binary cross entropy |
